# Supplementary material for: Patterns of Intron Gain and Loss in Fungi
Source: PLoS Biol. 2004 Nov 30;2(12):e422. doi: 10.1371/journal.pbio.0020422 (PMC532390; doi:10.1371/journal.pbio.0020422)
Supplement: Table S1 — Also available at http://genes.mit.edu/NielsenEtAl/. (4.3 MB ZIP). [file pbio.0020422.st001.zip › NielsenEtAl/html/991.html]

AN0814.1.NCU02616.1.MG01236.1.FG00660.1


```
 CLUSTAL W (1.82) Multiple Sequence Alignments - Introns Inserted


Sequence 1: MG01236.1	593 aa
Sequence 2: FG00660.1	597 aa
Sequence 3: NCU02616.1	603 aa
Sequence 4: AN0814.1	618 aa
Alignment Length: 625 aa
Number Identitical Residues: 379 aa
Alignment Score (without introns) 16541


MG01236.1 	MATAFCETPLKSHTGLFSSRTAGGRMPLTPSPRQRAVA-VAINVNSSPCTPDET----YK
NCU02616.1	MATPSVTTPKKSHTGLFSSRTAGGRVPLTPSPHH------NANVNSSPFNKDKTSNDGQR
FG00660.1 	MASVTLSTPVKSHKGLFTARTAGGRMPLTPSPRQQASANIPINLNSSPFTPERQSSNDFK
AN0814.1  	MATPLVSTPVKTHHGLFSTKMAGGRMPLTPSPGGRRDS--IASNNSSPFTPPRLESESTK
          	**:    ** *:* ***::: ****:******     :    . **** .  . .... :

MG01236.1 	PAGKSSYTSKLAAHLAAKQ------QRDSPKSNIARGVSTPRKALELGVSDFTLTGTGHQ
NCU02616.1	APAKTVYPGNISSQLARAASTKT--NWDSPKSNIARGASTPKKALELGVSEFTLTGTGAQ
FG00660.1 	PAGRSTYGGNLMAHLARSTTSA---HRDSPKSNIARVVHTPRKALELGVSDYTLTGTG--
AN0814.1  	EQARSVYGGNLSSYFAKSVSRSSRAYRESPKSNIARVRKSP-KHLELGVSEWSLTGTGPS
          	  .:: * .:: : :*   :  : :  :********   :* * ******:::***** .

MG01236.1 	ATKTPSSTKT----RKTIRQKTSKTSVSYAAGDRFIPNRTASSAIANVGSGKLDMHER-R
NCU02616.1	AQEQTQTAPTPKPKKTKIKAKPIKTTIALNA-DRFIPNRGSSSAIANGGSGKLNMRDQKR
FG00660.1 	SSKTPAN-------KKGIRSKVAKTTVNYAG-DRFIPNRGASSAISNAGSGKLDVPDRQR
AN0814.1  	SNQTPPKERLRKEVPSRARAGKTTVRISHNAGDRFIPNRSASEGLVTAGAAKP--EETQR
          	: : . .    .   .  :    .. :   ..******* :*..: . *:.*    : .*

MG01236.1 	PKSSSSESSG--VLSSAA-----------DEALAALEALNIGDDDDEAEPYSRPSPNTAA
NCU02616.1	PKSRNGESSNSTVLATPA-----------DDATEQLEFDSLLLDDDEAETYARPSPNTVA
FG00660.1 	PKTSTVESSN--VLSTAA-----------DEALAALESLNINDDD-EPDNYSRPSPNTVA
AN0814.1  	PKTSSGDKGSS-VLASAASAFDIGGRGTDEDLTAALENLGLDDDD-NSSSYSKPAPDAVA
          	**: . :...: **::.*:: . .. .:.::    **  .:  ** :.. *::*:*::.*

MG01236.1 	YQNSIANACGVSLNTRILEFKPAPPESSKPIDLRQQYNRPLKPASSSSAQSRRRIATAPE
NCU02616.1	YQDSLANACGMNVNTRILQFKPAPPESSKPIDLRQQYNRPLKAAN-SSAQFRRRVATAPE
FG00660.1 	YQDSLANACGVSLNTRILEFKPAAPESSKPIDLRQQYNRPLRSTTTTSAQIRRRIATAPE
AN0814.1  	YESSLANACGVNLNTRILAFKPPPPESSKPIDLRAQYNRPLKSTKSNSAQFRRRVQTAPE
          	*:.*:*****:.:***** ***..********** ******:.:.:.*** ***: ****

MG01236.1 	RVLDAPGLIDDYYLNLLDWSSGNQVAIGLERSVYVWSADEGSVSCLLETSPDTYVSSVKW
NCU02616.1	RVLDAPGLIDDYYLNLLDWSSGNQVAIGLERNVYVWSADEGSVNCLLETSPDTYVSSVKW
FG00660.1 	RVLDAPGLVDDYYLNLLDWSSGNQVAIGLERNVYVWSADEGSVSCLLETTPDTYVSSVKW
AN0814.1  	RVLDAPGLLDDYYLNLLDWSSGNQVAIGLERNVYVWSADSGSVSCLLETSPDTYISSVKW
          	********:**********************.*******.***.*****:****:*****

MG01236.1 	SADGAYVSVGMGTGEVQIWDVAEGAKVRSMHGHDTRVSVMGWNKHLLSTGARSGLVFNHD
NCU02616.1	SGDGAYVGVGLGTGEVQIWDVAEGIKIRSMFGHDSRVGVMGWSKHLLSTGARSGLVFNHD
FG00660.1 	SGDGAYVSVGLGTGEVQIWDVAEGQKIRSMFGHDTRVGVMGWNKHLLSTGARSGLVYNHD
AN0814.1  	SGDGAYVGVGLGTGEVQIWDVEEGTKLRSMFGHESRVGVMGWSKHTLSTGARSGLVFNHD
          	*.*****.**:********** ** *:***.**::**.****.** **********:***

MG01236.1 	VRIAEHKVAELVSHTSEVCGLEWRSDGAQLATGGNDNLVSIWDARSLAVPKFTKTNHKAA
NCU02616.1	VRIAEHKVAELVSHTSEVCGLEWRSDGAQLATGGNDNLVSIWDARSLAVPKFTKTNHKAA
FG00660.1 	VRIAEHKVAELISHTSEVCGLEWRSDGAQLATGGNDNLVSIWDARSLSVPKFTKTNHKAA
AN0814.1  	VRIAQHKVAELVSHTSEVCGLEWRSDGAQLATGGNDNLVNIWDARSLSAPKFTKTNHRAA
          	****:******:***************************.*******:.********:**

MG01236.1 	VKALAWCPWNMNLLATGGGSYDRHIHFWNSTSGARVNSIDTGSQVTSLRWSPHYREIVSS
NCU02616.1	VKALAWCPWNINLLATGGGAYDRHIHFWNTTSGARVNSIDTGSQVTSLRWSPRYREIVSS
FG00660.1 	VKALSWCPWNMNLLATGGGSYDRHIHFWNSTSGARVNSIDTGSQVTSLRWSPHHREIVSS
AN0814.1  	VKALSWCPWQLNLLATGGGSYDRHIHFWNTTTGARTNSIDTGSQVTSLRWSNHYREIVSS
          	****:****::********:*********:*:***.*************** ::******

MG01236.1 	SGFPDNSLSIWSYPTLVRTVEIPAHESRVLHSCLSPDGQMLATAA~ADESLKFWKVFEKK
NCU02616.1	SGYPDNSLSIWSYPTLVRNVEIPAHESRVLHSCLSPDGQMLATAA1ADESLKFWKIFEKK
FG00660.1 	SGFPDNSLSIWSYPTLVRTVEIPAHESRVLHSCLSPDGQMLATAA1ADESLKFWKIFEKK
AN0814.1  	SGFPDNSLSIWSYPTLVRNIEIPAHETRVLHSCLSPDGQLLATAA~ADESLKFWKVFERK
          	**:***************.:******:************:***** *********:**:*

MG01236.1 	PGASS--AGLGAS-SKADMVKQMTIR
NCU02616.1	AGSGAGISGAGTS-SKATMAKQMTIR
FG00660.1 	AGATASIGSSGAS-SKASMAKQMTIR
AN0814.1  	PGTSASASREGGVGSKAQMTKSMTIR
          	.*: :. .  *  .*** *.*.****
```
